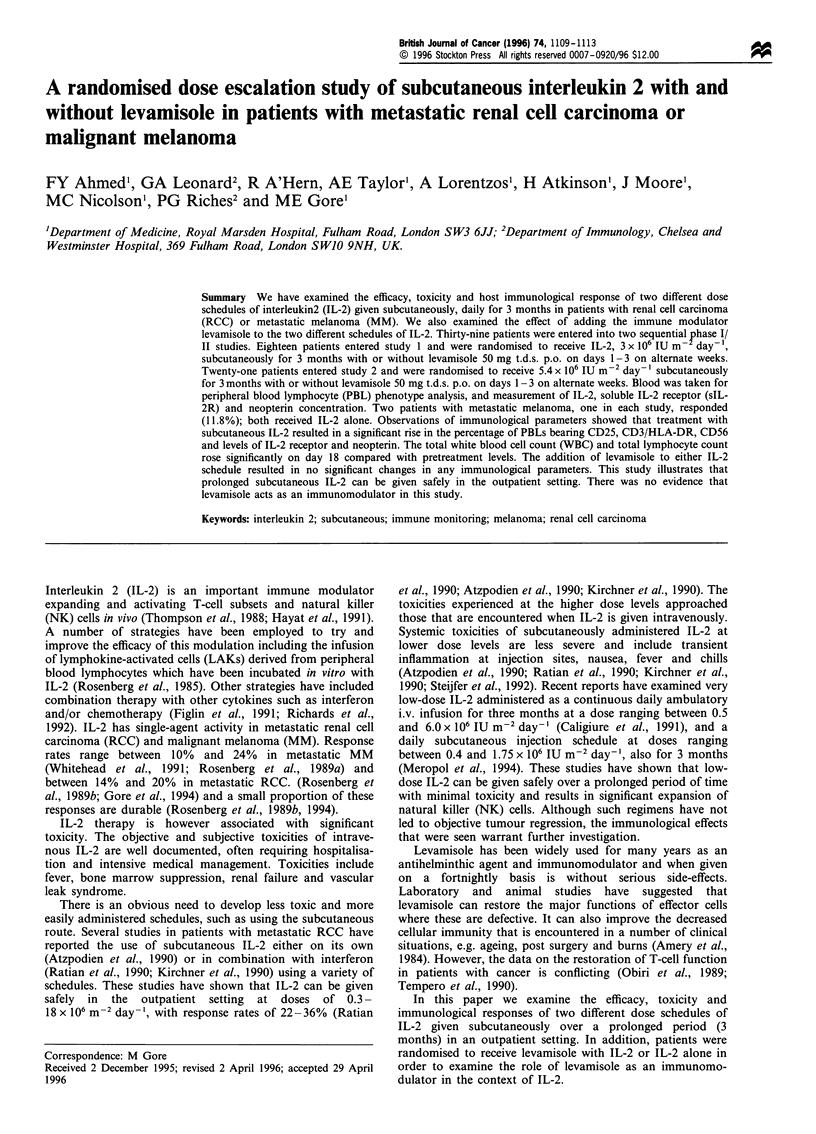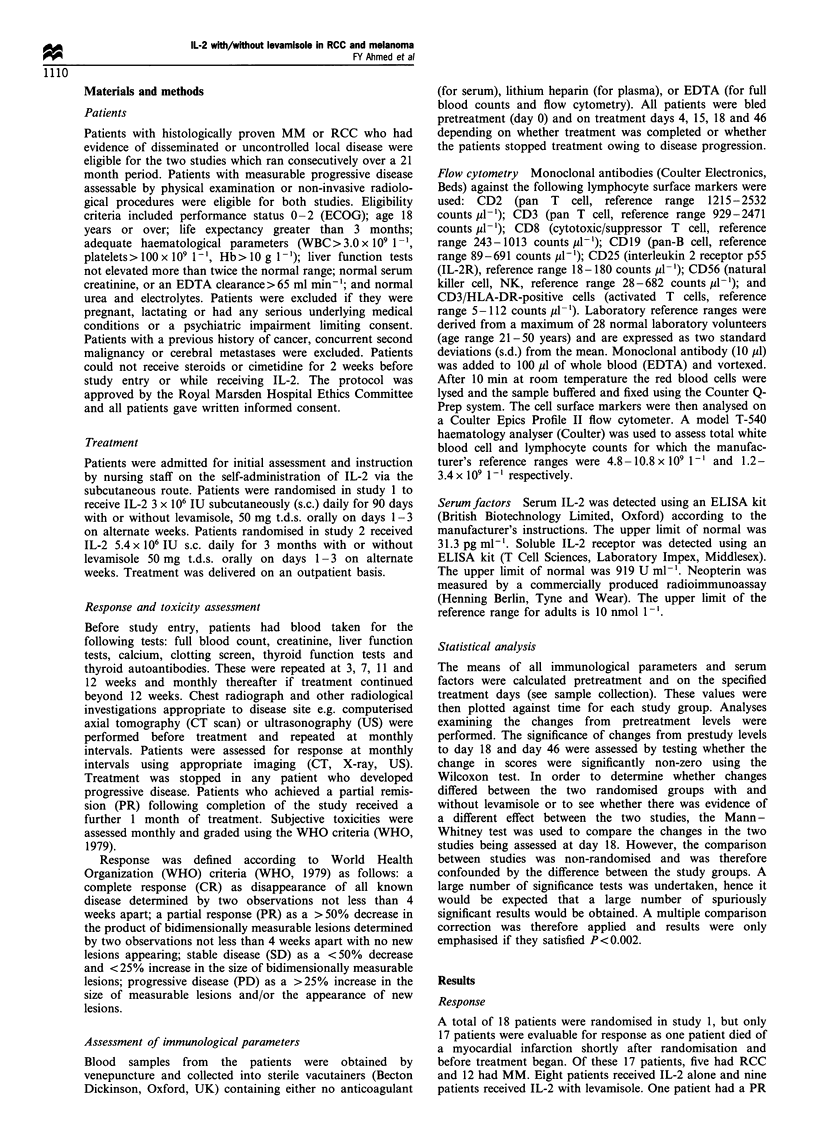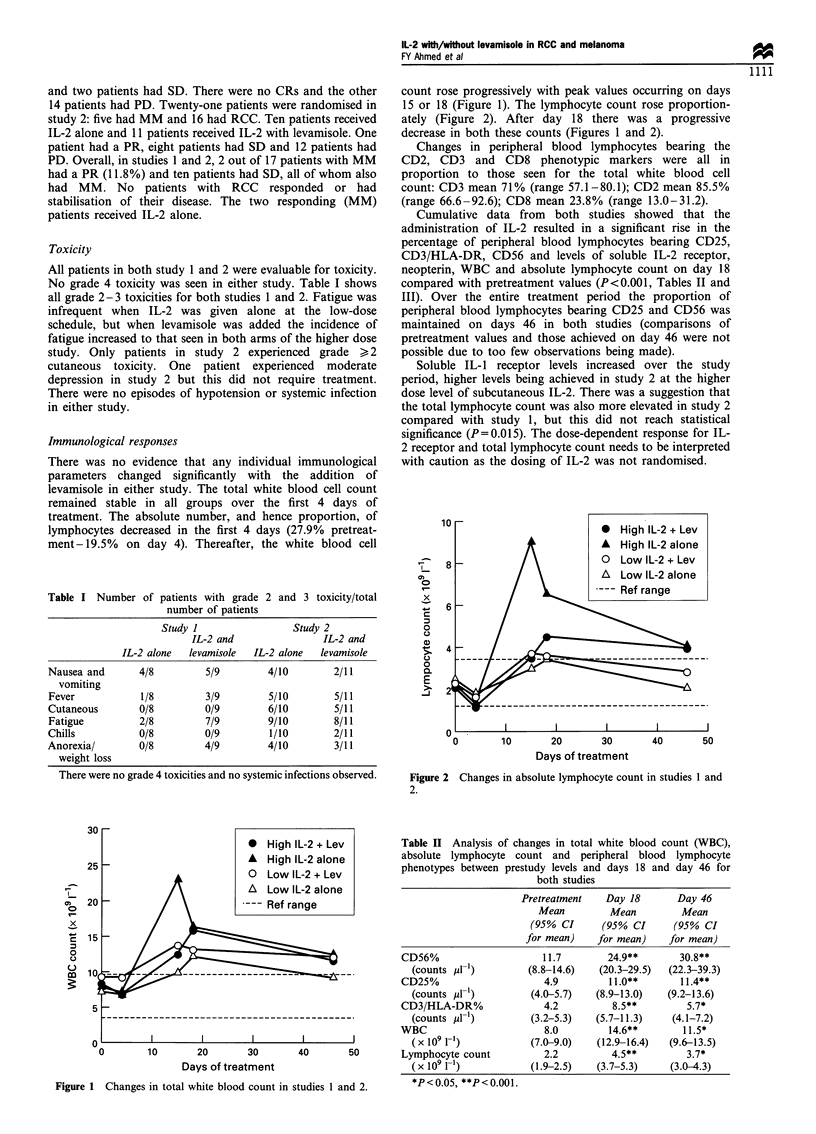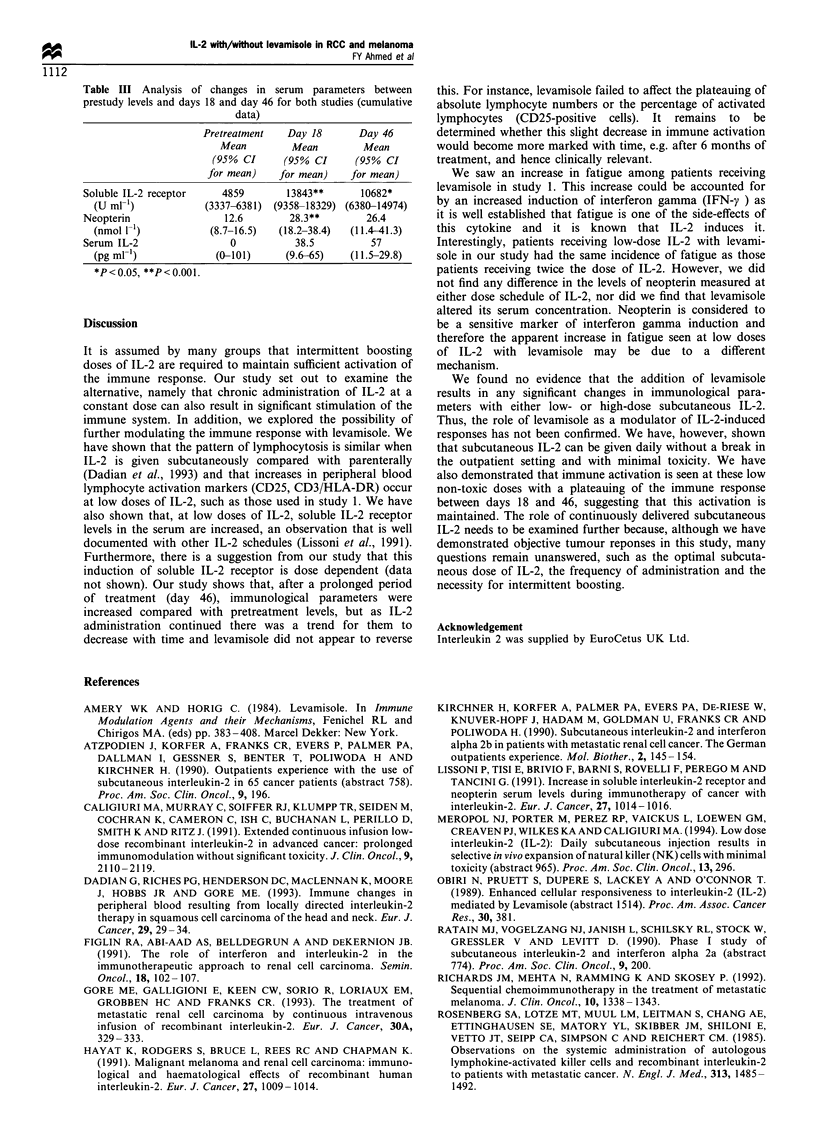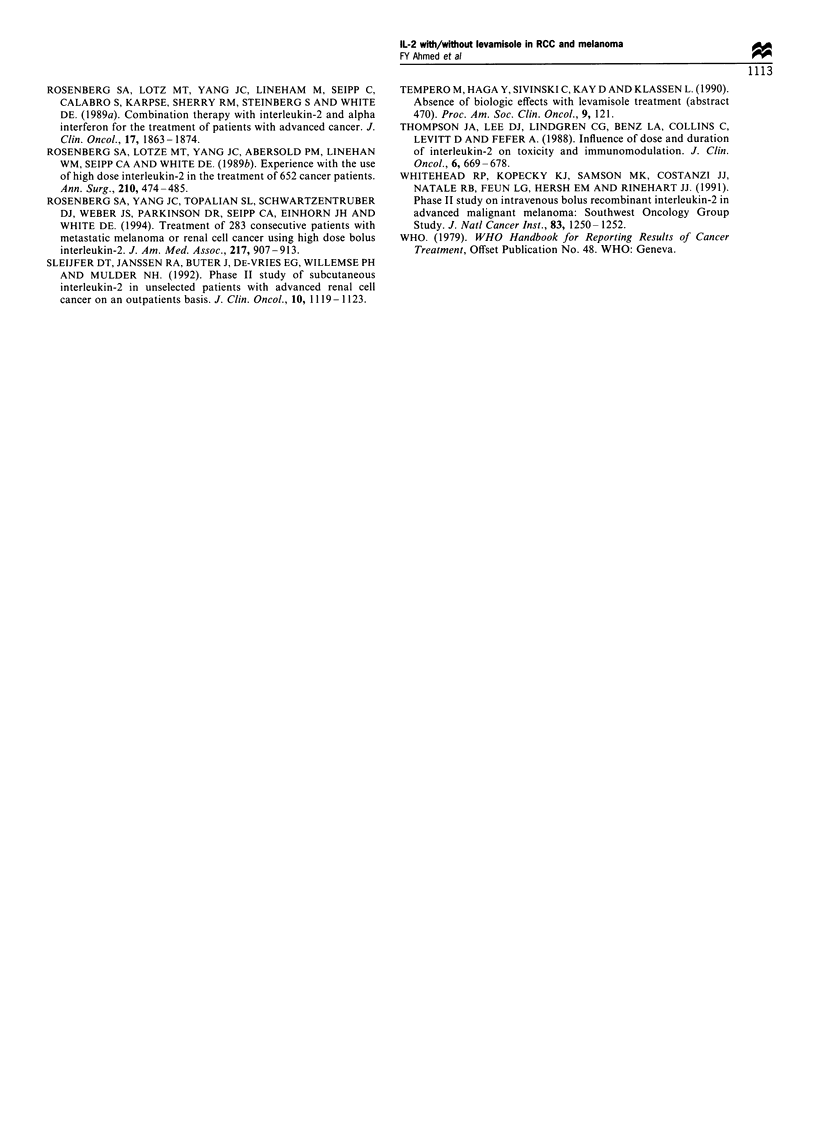# A randomised dose escalation study of subcutaneous interleukin 2 with and without levamisole in patients with metastatic renal cell carcinoma or malignant melanoma.

**DOI:** 10.1038/bjc.1996.498

**Published:** 1996-10

**Authors:** F. Y. Ahmed, G. A. Leonard, R. A'Hern, A. E. Taylor, A. Lorentzos, H. Atkinson, J. Moore, M. C. Nicolson, P. G. Riches, M. E. Gore

**Affiliations:** Department of Medicine, Royal Marsden Hospital, London, UK.

## Abstract

We have examined the efficacy, toxicity and host immunological response of two different dose schedules of interleukin 2 (IL-2) given subcutaneously, daily for 3 months in patients with renal cell carcinoma (RCC) or metastatic melanoma (MM). We also examined the effect of adding the immune modulator levamisole to the two different schedules of IL-2. Thirty-nine patients were entered into two sequential phase I/II studies. Eighteen patients entered study 1 and were randomised to receive IL-2, 3 x 10(6) IU m-2 day-1, subcutaneously for 3 months with or without levamisole 50 mg t.d.s. p.o. on days 1-3 on alternate weeks. Twenty-one patients entered study 2 and were randomised to receive 5.4 x 10(6) IU m-2 day-1 subcutaneously for 3 months with or without levamisole 50 mg t.d.s. p.o. on days 1-3 on alternate weeks. Blood was taken for peripheral blood lymphocyte (PBL) phenotype analysis, and measurement of IL-2, soluble IL-2 receptor (sIL-2R) and neopterin concentration. Two patients with metastatic melanoma, one in each study, responded (11.8%); both received IL-2 alone. Observations of immunological parameters showed that treatment with subcutaneous IL-2 resulted in a significant rise in the percentage of PBLs bearing CD25, CD3/HLA-DR, CD56 and levels of IL-2 receptor and neopterin. The total white blood cell count (WBC) and total lymphocyte count rose significantly on day 18 compared with pretreatment levels. The addition of levamisole to either IL-2 schedule resulted in no significant changes in any immunological parameters. This study illustrates that prolonged subcutaneous IL-2 can be given safely in the outpatient setting. There was no evidence that levamisole acts as an immunomodulator in this study.


British Journal of Cancer (1996) 74, 1109-1113
© 1996 Stockton Press All rights reserved 0007-0920/96 $12.00

### A randomised dose escalation study of subcutaneous interleukin 2 with and without levamisole in patients with metastatic renal cell carcinoma or malignant melanoma

FY Ahmed[1], GA Leonard[2], R A'Hern, AE Taylor[1], A Lorentzos[1], H Atkinson[1], J Moore[1], MC Nicolson[1], PG Riches[2] and ME Gore[1]

[1]*Department of Medicine, Royal Marsden Hospital, Fulham Road, London SW3 6JJ;* [2]*Department of Immunology, Chelsea and Westminster Hospital, 369 Fulham Road, London SW10 9NH, UK.*

**Summary** We have examined the efficacy, toxicity and host immunological response of two different dose schedules of interleukin2 (IL-2) given subcutaneously, daily for 3 months in patients with renal cell carcinoma (RCC) or metastatic melanoma (MM). We also examined the effect of adding the immune modulator levamisole to the two different schedules of IL-2. Thirty-nine patients were entered into two sequential phase I/II studies. Eighteen patients entered study 1 and were randomised to receive IL-2, $3 \times 10^6$ IU $m^{-2}$ $day^{-1}$, subcutaneously for 3 months with or without levamisole 50 mg t.d.s. p.o. on days 1-3 on alternate weeks. Twenty-one patients entered study 2 and were randomised to receive $5.4 \times 10^6$ IU $m^{-2}$ $day^{-1}$ subcutaneously for 3 months with or without levamisole 50 mg t.d.s. p.o. on days 1-3 on alternate weeks. Blood was taken for peripheral blood lymphocyte (PBL) phenotype analysis, and measurement of IL-2, soluble IL-2 receptor (sIL-2R) and neopterin concentration. Two patients with metastatic melanoma, one in each study, responded (11.8%); both received IL-2 alone. Observations of immunological parameters showed that treatment with subcutaneous IL-2 resulted in a significant rise in the percentage of PBLs bearing CD25, CD3/HLA-DR, CD56 and levels of IL-2 receptor and neopterin. The total white blood cell count (WBC) and total lymphocyte count rose significantly on day 18 compared with pretreatment levels. The addition of levamisole to either IL-2 schedule resulted in no significant changes in any immunological parameters. This study illustrates that prolonged subcutaneous IL-2 can be given safely in the outpatient setting. There was no evidence that levamisole acts as an immunomodulator in this study.

**Keywords:** interleukin 2; subcutaneous; immune monitoring; melanoma; renal cell carcinoma

Interleukin 2 (IL-2) is an important immune modulator expanding and activating T-cell subsets and natural killer (NK) cells *in vivo* (Thompson *et al.*, 1988; Hayat *et al.*, 1991). A number of strategies have been employed to try and improve the efficacy of this modulation including the infusion of lymphokine-activated cells (LAKs) derived from peripheral blood lymphocytes which have been incubated *in vitro* with IL-2 (Rosenberg *et al.*, 1985). Other strategies have included combination therapy with other cytokines such as interferon and/or chemotherapy (Figlin *et al.*, 1991; Richards *et al.*, 1992). IL-2 has single-agent activity in metastatic renal cell carcinoma (RCC) and malignant melanoma (MM). Response rates range between 10% and 24% in metastatic MM (Whitehead *et al.*, 1991; Rosenberg *et al.*, 1989*a*) and between 14% and 20% in metastatic RCC. (Rosenberg *et al.*, 1989*b*; Gore *et al.*, 1994) and a small proportion of these responses are durable (Rosenberg *et al.*, 1989*b*, 1994).

IL-2 therapy is however associated with significant toxicity. The objective and subjective toxicities of intravenous IL-2 are well documented, often requiring hospitalisation and intensive medical management. Toxicities include fever, bone marrow suppression, renal failure and vascular leak syndrome.

There is an obvious need to develop less toxic and more easily administered schedules, such as using the subcutaneous route. Several studies in patients with metastatic RCC have reported the use of subcutaneous IL-2 either on its own (Atzpodien *et al.*, 1990) or in combination with interferon (Ratian *et al.*, 1990; Kirchner *et al.*, 1990) using a variety of schedules. These studies have shown that IL-2 can be given safely in the outpatient setting at doses of $0.3-18 \times 10^6$ $m^{-2}$ $day^{-1}$, with response rates of 22-36% (Ratian *et al.*, 1990; Atzpodien *et al.*, 1990; Kirchner *et al.*, 1990). The toxicities experienced at the higher dose levels approached those that are encountered when IL-2 is given intravenously. Systemic toxicities of subcutaneously administered IL-2 at lower dose levels are less severe and include transient inflammation at injection sites, nausea, fever and chills (Atzpodien *et al.*, 1990; Ratian *et al.*, 1990; Kirchner *et al.*, 1990; Steijfer *et al.*, 1992). Recent reports have examined very low-dose IL-2 administered as a continuous daily ambulatory i.v. infusion for three months at a dose ranging between 0.5 and $6.0 \times 10^6$ IU $m^{-2}$ $day^{-1}$ (Caligiure *et al.*, 1991), and a daily subcutaneous injection schedule at doses ranging between 0.4 and $1.75 \times 10^6$ IU $m^{-2}$ $day^{-1}$, also for 3 months (Meropol *et al.*, 1994). These studies have shown that low-dose IL-2 can be given safely over a prolonged period of time with minimal toxicity and results in significant expansion of natural killer (NK) cells. Although such regimens have not led to objective tumour regression, the immunological effects that were seen warrant further investigation.

Levamisole has been widely used for many years as an antihelminthic agent and immunomodulator and when given on a fortnightly basis is without serious side-effects. Laboratory and animal studies have suggested that levamisole can restore the major functions of effector cells where these are defective. It can also improve the decreased cellular immunity that is encountered in a number of clinical situations, e.g. ageing, post surgery and burns (Amery *et al.*, 1984). However, the data on the restoration of T-cell function in patients with cancer is conflicting (Obiri *et al.*, 1989; Tempero *et al.*, 1990).

In this paper we examine the efficacy, toxicity and immunological responses of two different dose schedules of IL-2 given subcutaneously over a prolonged period (3 months) in an outpatient setting. In addition, patients were randomised to receive levamisole with IL-2 or IL-2 alone in order to examine the role of levamisole as an immunomodulator in the context of IL-2.

Correspondence: M Gore
Received 2 December 1995; revised 2 April 1996; accepted 29 April 1996

### Materials and methods

### Patients

Patients with histologically proven MM or RCC who had evidence of disseminated or uncontrolled local disease were eligible for the two studies which ran consecutively over a 21 month period. Patients with measurable progressive disease assessable by physical examination or non-invasive radiological procedures were eligible for both studies. Eligibility criteria included performance status 0–2 (ECOG); age 18 years or over; life expectancy greater than 3 months; adequate haematological parameters (WBC > $3.0 \times 10^9$ $1^{-1}$, platelets > $100 \times 10^9$ $1^{-1}$, Hb > 10 g $1^{-1}$); liver function tests not elevated more than twice the normal range; normal serum creatinine, or an EDTA clearance > 65 ml $min^{-1}$; and normal urea and electrolytes. Patients were excluded if they were pregnant, lactating or had any serious underlying medical conditions or a psychiatric impairment limiting consent. Patients with a previous history of cancer, concurrent second malignancy or cerebral metastases were excluded. Patients could not receive steroids or cimetidine for 2 weeks before study entry or while receiving IL-2. The protocol was approved by the Royal Marsden Hospital Ethics Committee and all patients gave written informed consent.

### Treatment

Patients were admitted for initial assessment and instruction by nursing staff on the self-administration of IL-2 via the subcutaneous route. Patients were randomised in study 1 to receive IL-2 $3 \times 10^6$ IU subcutaneously (s.c.) daily for 90 days with or without levamisole, 50 mg t.d.s. orally on days 1–3 on alternate weeks. Patients randomised in study 2 received IL-2 $5.4 \times 10^6$ IU s.c. daily for 3 months with or without levamisole 50 mg t.d.s. orally on days 1–3 on alternate weeks. Treatment was delivered on an outpatient basis.

### Response and toxicity assessment

Before study entry, patients had blood taken for the following tests: full blood count, creatinine, liver function tests, calcium, clotting screen, thyroid function tests and thyroid autoantibodies. These were repeated at 3, 7, 11 and 12 weeks and monthly thereafter if treatment continued beyond 12 weeks. Chest radiograph and other radiological investigations appropriate to disease site e.g. computerised axial tomography (CT scan) or ultrasonography (US) were performed before treatment and repeated at monthly intervals. Patients were assessed for response at monthly intervals using appropriate imaging (CT, X-ray, US). Treatment was stopped in any patient who developed progressive disease. Patients who achieved a partial remission (PR) following completion of the study received a further 1 month of treatment. Subjective toxicities were assessed monthly and graded using the WHO criteria (WHO, 1979).

Response was defined according to World Health Organization (WHO) criteria (WHO, 1979) as follows: a complete response (CR) as disappearance of all known disease determined by two observations not less than 4 weeks apart; a partial response (PR) as a > 50% decrease in the product of bidimensionally measurable lesions determined by two observations not less than 4 weeks apart with no new lesions appearing; stable disease (SD) as a < 50% decrease and < 25% increase in the size of bidimensionally measurable lesions; progressive disease (PD) as a > 25% increase in the size of measurable lesions and/or the appearance of new lesions.

### Assessment of immunological parameters

Blood samples from the patients were obtained by venepuncture and collected into sterile vacutainers (Becton Dickinson, Oxford, UK) containing either no anticoagulant (for serum), lithium heparin (for plasma), or EDTA (for full blood counts and flow cytometry). All patients were bled pretreatment (day 0) and on treatment days 4, 15, 18 and 46 depending on whether treatment was completed or whether the patients stopped treatment owing to disease progression.

*Flow cytometry* Monoclonal antibodies (Coulter Electronics, Beds) against the following lymphocyte surface markers were used: CD2 (pan T cell, reference range 1215–2532 counts $\mu l^{-1}$); CD3 (pan T cell, reference range 929–2471 counts $\mu l^{-1}$); CD8 (cytotoxic/suppressor T cell, reference range 243–1013 counts $\mu l^{-1}$); CD19 (pan-B cell, reference range 89–691 counts $\mu l^{-1}$); CD25 (interleukin 2 receptor p55 (IL-2R), reference range 18–180 counts $\mu l^{-1}$); CD56 (natural killer cell, NK, reference range 28–682 counts $\mu l^{-1}$); and CD3/HLA-DR-positive cells (activated T cells, reference range 5–112 counts $\mu l^{-1}$). Laboratory reference ranges were derived from a maximum of 28 normal laboratory volunteers (age range 21–50 years) and are expressed as two standard deviations (s.d.) from the mean. Monoclonal antibody (10 $\mu l$) was added to 100 $\mu l$ of whole blood (EDTA) and vortexed. After 10 min at room temperature the red blood cells were lysed and the sample buffered and fixed using the Counter Q-Prep system. The cell surface markers were then analysed on a Coulter Epics Profile II flow cytometer. A model T-540 haematology analyser (Coulter) was used to assess total white blood cell and lymphocyte counts for which the manufacturer's reference ranges were $4.8-10.8 \times 10^9$ $1^{-1}$ and $1.2-3.4 \times 10^9$ $1^{-1}$ respectively.

*Serum factors* Serum IL-2 was detected using an ELISA kit (British Biotechnology Limited, Oxford) according to the manufacturer's instructions. The upper limit of normal was 31.3 pg $ml^{-1}$. Soluble IL-2 receptor was detected using an ELISA kit (T Cell Sciences, Laboratory Impex, Middlesex). The upper limit of normal was 919 U $ml^{-1}$. Neopterin was measured by a commercially produced radioimmunoassay (Henning Berlin, Tyne and Wear). The upper limit of the reference range for adults is 10 nmol $1^{-1}$.

### Statistical analysis

The means of all immunological parameters and serum factors were calculated pretreatment and on the specified treatment days (see sample collection). These values were then plotted against time for each study group. Analyses examining the changes from pretreatment levels were performed. The significance of changes from prestudy levels to day 18 and day 46 were assessed by testing whether the change in scores were significantly non-zero using the Wilcoxon test. In order to determine whether changes differed between the two randomised groups with and without levamisole or to see whether there was evidence of a different effect between the two studies, the Mann–Whitney test was used to compare the changes in the two studies being assessed at day 18. However, the comparison between studies was non-randomised and was therefore confounded by the difference between the study groups. A large number of significance tests was undertaken, hence it would be expected that a large number of spuriously significant results would be obtained. A multiple comparison correction was therefore applied and results were only emphasised if they satisfied $P < 0.002$.

### Results

### Response

A total of 18 patients were randomised in study 1, but only 17 patients were evaluable for response as one patient died of a myocardial infarction shortly after randomisation and before treatment began. Of these 17 patients, five had RCC and 12 had MM. Eight patients received IL-2 alone and nine patients received IL-2 with levamisole. One patient had a PR

and two patients had SD. There were no CRs and the other 14 patients had PD. Twenty-one patients were randomised in study 2: five had MM and 16 had RCC. Ten patients received IL-2 alone and 11 patients received IL-2 with levamisole. One patient had a PR, eight patients had SD and 12 patients had PD. Overall, in studies 1 and 2, 2 out of 17 patients with MM had a PR (11.8%) and ten patients had SD, all of whom also had MM. No patients with RCC responded or had stabilisation of their disease. The two responding (MM) patients received IL-2 alone.

*Toxicity*

All patients in both study 1 and 2 were evaluable for toxicity. No grade 4 toxicity was seen in either study. Table I shows all grade 2–3 toxicities for both studies 1 and 2. Fatigue was infrequent when IL-2 was given alone at the low-dose schedule, but when levamisole was added the incidence of fatigue increased to that seen in both arms of the higher dose study. Only patients in study 2 experienced grade ⩾2 cutaneous toxicity. One patient experienced moderate depression in study 2 but this did not require treatment. There were no episodes of hypotension or systemic infection in either study.

*Immunological responses*

There was no evidence that any individual immunological parameters changed significantly with the addition of levamisole in either study. The total white blood cell count remained stable in all groups over the first 4 days of treatment. The absolute number, and hence proportion, of lymphocytes decreased in the first 4 days (27.9% pretreatment–19.5% on day 4). Thereafter, the white blood cell count rose progressively with peak values occurring on days 15 or 18 (Figure 1). The lymphocyte count rose proportionately (Figure 2). After day 18 there was a progressive decrease in both these counts (Figures 1 and 2).

Changes in peripheral blood lymphocytes bearing the CD2, CD3 and CD8 phenotypic markers were all in proportion to those seen for the total white blood cell count: CD3 mean 71% (range 57.1–80.1); CD2 mean 85.5% (range 66.6–92.6); CD8 mean 23.8% (range 13.0–31.2).

Cumulative data from both studies showed that the administration of IL-2 resulted in a significant rise in the percentage of peripheral blood lymphocytes bearing CD25, CD3/HLA-DR, CD56 and levels of soluble IL-2 receptor, neopterin, WBC and absolute lymphocyte count on day 18 compared with pretreatment values ($P<0.001$, Tables II and III). Over the entire treatment period the proportion of peripheral blood lymphocytes bearing CD25 and CD56 was maintained on days 46 in both studies (comparisons of pretreatment values and those achieved on day 46 were not possible due to too few observations being made).

Soluble IL-1 receptor levels increased over the study period, higher levels being achieved in study 2 at the higher dose level of subcutaneous IL-2. There was a suggestion that the total lymphocyte count was also more elevated in study 2 compared with study 1, but this did not reach statistical significance ($P=0.015$). The dose-dependent response for IL-2 receptor and total lymphocyte count needs to be interpreted with caution as the dosing of IL-2 was not randomised.

**Table I** Number of patients with grade 2 and 3 toxicity/total number of patients

| | *Study 1* | | *Study 2* | |
|---|---|---|---|---|
| | *IL-2 alone* | *IL-2 and levamisole* | *IL-2 alone* | *IL-2 and levamisole* |
| Nausea and vomiting | 4/8 | 5/9 | 4/10 | 2/11 |
| Fever | 1/8 | 3/9 | 5/10 | 5/11 |
| Cutaneous | 0/8 | 0/9 | 6/10 | 5/11 |
| Fatigue | 2/8 | 7/9 | 9/10 | 8/11 |
| Chills | 0/8 | 0/9 | 1/10 | 2/11 |
| Anorexia/ weight loss | 0/8 | 4/9 | 4/10 | 3/11 |

There were no grade 4 toxicities and no systemic infections observed.

**Figure 2** Changes in absolute lymphocyte count in studies 1 and 2.

**Figure 1** Changes in total white blood count in studies 1 and 2.

**Table II** Analysis of changes in total white blood count (WBC), absolute lymphocyte count and peripheral blood lymphocyte phenotypes between prestudy levels and days 18 and day 46 for both studies

| | *Pretreatment Mean (95% CI for mean)* | *Day 18 Mean (95% CI for mean)* | *Day 46 Mean (95% CI for mean)* |
|---|---|---|---|
| CD56% | 11.7 | 24.9** | 30.8** |
| (counts $\mu l^{-1}$) | (8.8–14.6) | (20.3–29.5) | (22.3–39.3) |
| CD25% | 4.9 | 11.0** | 11.4** |
| (counts $\mu l^{-1}$) | (4.0–5.7) | (8.9–13.0) | (9.2–13.6) |
| CD3/HLA-DR% | 4.2 | 8.5** | 5.7* |
| (counts $\mu l^{-1}$) | (3.2–5.3) | (5.7–11.3) | (4.1–7.2) |
| WBC | 8.0 | 14.6** | 11.5* |
| ($\times 10^9$ $l^{-1}$) | (7.0–9.0) | (12.9–16.4) | (9.6–13.5) |
| Lymphocyte count | 2.2 | 4.5** | 3.7* |
| ($\times 10^9$ $l^{-1}$) | (1.9–2.5) | (3.7–5.3) | (3.0–4.3) |

*$P<0.05$, **$P<0.001$.

**Table III** Analysis of changes in serum parameters between prestudy levels and days 18 and day 46 for both studies (cumulative data)

| | *Pretreatment Mean (95% CI for mean)* | *Day 18 Mean (95% CI for mean)* | *Day 46 Mean (95% CI for mean)* |
|---|---|---|---|
| Soluble IL-2 receptor | 4859 | 13843** | 10682* |
| (U $ml^{-1}$) | (3337–6381) | (9358–18329) | (6380–14974) |
| Neopterin | 12.6 | 28.3** | 26.4 |
| (nmol $l^{-1}$) | (8.7–16.5) | (18.2–38.4) | (11.4–41.3) |
| Serum IL-2 | 0 | 38.5 | 57 |
| (pg $ml^{-1}$) | (0–101) | (9.6–65) | (11.5–29.8) |

*$P<0.05$, **$P<0.001$.

### Discussion

It is assumed by many groups that intermittent boosting doses of IL-2 are required to maintain sufficient activation of the immune response. Our study set out to examine the alternative, namely that chronic administration of IL-2 at a constant dose can also result in significant stimulation of the immune system. In addition, we explored the possibility of further modulating the immune response with levamisole. We have shown that the pattern of lymphocytosis is similar when IL-2 is given subcutaneously compared with parenterally (Dadian *et al.*, 1993) and that increases in peripheral blood lymphocyte activation markers (CD25, CD3/HLA-DR) occur at low doses of IL-2, such as those used in study 1. We have also shown that, at low doses of IL-2, soluble IL-2 receptor levels in the serum are increased, an observation that is well documented with other IL-2 schedules (Lissoni *et al.*, 1991). Furthermore, there is a suggestion from our study that this induction of soluble IL-2 receptor is dose dependent (data not shown). Our study shows that, after a prolonged period of treatment (day 46), immunological parameters were increased compared with pretreatment levels, but as IL-2 administration continued there was a trend for them to decrease with time and levamisole did not appear to reverse this. For instance, levamisole failed to affect the plateauing of absolute lymphocyte numbers or the percentage of activated lymphocytes (CD25-positive cells). It remains to be determined whether this slight decrease in immune activation would become more marked with time, e.g. after 6 months of treatment, and hence clinically relevant.

We saw an increase in fatigue among patients receiving levamisole in study 1. This increase could be accounted for by an increased induction of interferon gamma (IFN-$\gamma$ ) as it is well established that fatigue is one of the side-effects of this cytokine and it is known that IL-2 induces it. Interestingly, patients receiving low-dose IL-2 with levamisole in our study had the same incidence of fatigue as those patients receiving twice the dose of IL-2. However, we did not find any difference in the levels of neopterin measured at either dose schedule of IL-2, nor did we find that levamisole altered its serum concentration. Neopterin is considered to be a sensitive marker of interferon gamma induction and therefore the apparent increase in fatigue seen at low doses of IL-2 with levamisole may be due to a different mechanism.

We found no evidence that the addition of levamisole results in any significant changes in immunological parameters with either low- or high-dose subcutaneous IL-2. Thus, the role of levamisole as a modulator of IL-2-induced responses has not been confirmed. We have, however, shown that subcutaneous IL-2 can be given daily without a break in the outpatient setting and with minimal toxicity. We have also demonstrated that immune activation is seen at these low non-toxic doses with a plateauing of the immune response between days 18 and 46, suggesting that this activation is maintained. The role of continuously delivered subcutaneous IL-2 needs to be examined further because, although we have demonstrated objective tumour reponses in this study, many questions remain unanswered, such as the optimal subcutaneous dose of IL-2, the frequency of administration and the necessity for intermittent boosting.

**Acknowledgement**
Interleukin 2 was supplied by EuroCetus UK Ltd.

### References

AMERY WK AND HORIG C. (1984). Levamisole. In *Immune Modulation Agents and their Mechanisms*, Fenichel RL and Chirigos MA. (eds) pp. 383–408. Marcel Dekker: New York.

ATZPODIEN J, KORFER A, FRANKS CR, EVERS P, PALMER PA, DALLMAN I, GESSNER S, BENTER T, POLIWODA H AND KIRCHNER H. (1990). Outpatients experience with the use of subcutaneous interleukin-2 in 65 cancer patients (abstract 758). *Proc. Am. Soc. Clin. Oncol.*, **9**, 196.

CALIGIURI MA, MURRAY C, SOIFFER RJ, KLUMPP TR, SEIDEN M, COCHRAN K, CAMERON C, ISH C, BUCHANAN L, PERILLO D, SMITH K AND RITZ J. (1991). Extended continuous infusion low-dose recombinant interleukin-2 in advanced cancer: prolonged immunomodulation without significant toxicity. *J. Clin. Oncol.*, **9**, 2110–2119.

DADIAN G, RICHES PG, HENDERSON DC, MACLENNAN K, MOORE J, HOBBS JR AND GORE ME. (1993). Immune changes in peripheral blood resulting from locally directed interleukin-2 therapy in squamous cell carcinoma of the head and neck. *Eur. J. Cancer*, **29**, 29–34.

FIGLIN RA, ABI-AAD AS, BELLDEGRUN A AND DEKERNION JB. (1991). The role of interferon and interleukin-2 in the immunotherapeutic approach to renal cell carcinoma. *Semin. Oncol.*, **18**, 102–107.

GORE ME, GALLIGIONI E, KEEN CW, SORIO R, LORIAUX EM, GROBBEN HC AND FRANKS CR. (1993). The treatment of metastatic renal cell carcinoma by continuous intravenous infusion of recombinant interleukin-2. *Eur. J. Cancer*, **30A**, 329–333.

HAYAT K, RODGERS S, BRUCE L, REES RC AND CHAPMAN K. (1991). Malignant melanoma and renal cell carcinoma: immunological and haematological effects of recombinant human interleukin-2. *Eur. J. Cancer*, **27**, 1009–1014.

KIRCHNER H, KORFER A, PALMER PA, EVERS PA, DE-RIESE W, KNUVER-HOPF J, HADAM M, GOLDMAN U, FRANKS CR AND POLIWODA H. (1990). Subcutaneous interleukin-2 and interferon alpha 2b in patients with metastatic renal cell cancer. The German outpatients experience. *Mol. Biother.*, **2**, 145–154.

LISSONI P, TISI E, BRIVIO F, BARNI S, ROVELLI F, PEREGO M AND TANCINI G. (1991). Increase in soluble interleukin-2 receptor and neopterin serum levels during immunotherapy of cancer with interleukin-2. *Eur. J. Cancer*, **27**, 1014–1016.

MEROPOL NJ, PORTER M, PEREZ RP, VAICKUS L, LOEWEN GM, CREAVEN PJ, WILKES KA AND CALIGIURI MA. (1994). Low dose interleukin-2 (IL-2): Daily subcutaneous injection results in selective *in vivo* expansion of natural killer (NK) cells with minimal toxicity (abstract 965). *Proc. Am. Soc. Clin. Oncol.*, **13**, 296.

OBIRI N, PRUETT S, DUPERE S, LACKEY A AND O'CONNOR T. (1989). Enhanced cellular responsiveness to interleukin-2 (IL-2) mediated by Levamisole (abstract 1514). *Proc. Am. Assoc. Cancer Res.*, **30**, 381.

RATAIN MJ, VOGELZANG NJ, JANISH L, SCHILSKY RL, STOCK W, GRESSLER V AND LEVITT D. (1990). Phase I study of subcutaneous interleukin-2 and interferon alpha 2a (abstract 774). *Proc. Am. Soc. Clin. Oncol.*, **9**, 200.

RICHARDS JM, MEHTA N, RAMMING K AND SKOSEY P. (1992). Sequential chemoimmunotherapy in the treatment of metastatic melanoma. *J. Clin. Oncol.*, **10**, 1338–1343.

ROSENBERG SA, LOTZE MT, MUUL LM, LEITMAN S, CHANG AE, ETTINGHAUSEN SE, MATORY YL, SKIBBER JM, SHILONI E, VETTO JT, SEIPP CA, SIMPSON C AND REICHERT CM. (1985). Observations on the systemic administration of autologous lymphokine-activated killer cells and recombinant interleukin-2 to patients with metastatic cancer. *N. Engl. J. Med.*, **313**, 1485–1492.

ROSENBERG SA, LOTZ MT, YANG JC, LINEHAM M, SEIPP C, CALABRO S, KARPSE, SHERRY RM, STEINBERG S AND WHITE DE. (1989*a*). Combination therapy with interleukin-2 and alpha interferon for the treatment of patients with advanced cancer. *J. Clin. Oncol.*, **17,** 1863–1874.

ROSENBERG SA, LOTZE MT, YANG JC, ABERSOLD PM, LINEHAN WM, SEIPP CA AND WHITE DE. (1989*b*). Experience with the use of high dose interleukin-2 in the treatment of 652 cancer patients. *Ann. Surg.*, **210,** 474–485.

ROSENBERG SA, YANG JC, TOPALIAN SL, SCHWARTZENTRUBER DJ, WEBER JS, PARKINSON DR, SEIPP CA, EINHORN JH AND WHITE DE. (1994). Treatment of 283 consecutive patients with metastatic melanoma or renal cell cancer using high dose bolus interleukin-2. *J. Am. Med. Assoc.*, **217,** 907–913.

SLEIJFER DT, JANSSEN RA, BUTER J, DE-VRIES EG, WILLEMSE PH AND MULDER NH. (1992). Phase II study of subcutaneous interleukin-2 in unselected patients with advanced renal cell cancer on an outpatients basis. *J. Clin. Oncol.*, **10,** 1119–1123.

TEMPERO M, HAGA Y, SIVINSKI C, KAY D AND KLASSEN L. (1990). Absence of biologic effects with levamisole treatment (abstract 470). *Proc. Am. Soc. Clin. Oncol.*, **9,** 121.

THOMPSON JA, LEE DJ, LINDGREN CG, BENZ LA, COLLINS C, LEVITT D AND FEFER A. (1988). Influence of dose and duration of interleukin-2 on toxicity and immunomodulation. *J. Clin. Oncol.*, **6,** 669–678.

WHITEHEAD RP, KOPECKY KJ, SAMSON MK, COSTANZI JJ, NATALE RB, FEUN LG, HERSH EM AND RINEHART JJ. (1991). Phase II study on intravenous bolus recombinant interleukin-2 in advanced malignant melanoma: Southwest Oncology Group Study. *J. Natl Cancer Inst.*, **83,** 1250–1252.

WHO. (1979). *WHO Handbook for Reporting Results of Cancer Treatment*, Offset Publication No. 48. WHO: Geneva.